# Structural, Optical and Dielectric Properties of Some Nanocomposites Derived from Copper Oxide Nanoparticles Embedded in Poly(vinylpyrrolidone) Matrix

**DOI:** 10.3390/nano14090759

**Published:** 2024-04-25

**Authors:** Carmen Gherasim, Mihai Asandulesa, Nicusor Fifere, Florica Doroftei, Daniel Tîmpu, Anton Airinei

**Affiliations:** Petru Poni Institute of Macromolecular Chemistry, 41A, Grigore Ghica Voda Alley, 700487 Iasi, Romania; gherasim.carmen@icmpp.ro (C.G.); asandulesa.mihai@icmpp.ro (M.A.); fifere.nicusor@icmpp.ro (N.F.); florica.doroftei@icmpp.ro (F.D.); dtimpu@icmpp.ro (D.T.)

**Keywords:** PVP/CuO nanocomposites, CuO nanoparticles, optical properties, band gap energy, dielectric properties

## Abstract

Polymer nanocomposite films based on poly(vinyl pyrrolidone) incorporated with different amounts of copper oxide (CuO) nanoparticles were prepared by the solution casting technique. The PVP/CuO nanocomposites were analyzed by X-ray diffractometry (XRD), scanning electron microscopy, UV–Visible absorption spectroscopy and dielectric spectroscopy. The XRD analysis showed that the monoclinic structure of cupric oxide was maintained in the PVP host matrix. The key optical parameters, such as optical energy gap E_g_, Urbach energy E_U_, absorption coefficient and refractive index, were estimated based on the UV-Vis data. The optical characteristics of the nanocomposite films revealed that their transmittance and absorption were influenced by the addition of CuO nanoparticles in the PVP matrix. Incorporation of CuO nanoparticles into the PVP matrix led to a significant decrease in band gap energy and an increase in the refractive index. The dielectric and electrical behaviors of the PVP/CuO nanocomposites were analyzed over a frequency range between 10 Hz and 1 MHz. The effect of CuO loading on the dielectric parameters (dielectric constant and dielectric loss) of the metal oxide nanocomposites was also discussed.

## 1. Introduction

Polymer-based metal oxide nanocomposites have attracted increasing attention for obtaining new materials with tailored characteristics without the need to prepare totally new products. The properties of these materials can be controlled by varying the polymer matrix, nanoparticle nature and their composition. By including metal oxide nanoparticles in polymer matrices to create nanocomposites, it is possible to enhance the performance of these materials, such as structural, electrical, optical and mechanical properties, due to the interaction between the polymer matrix and the nanoparticle component [1,2,3,4,5].

Polymer-based metal oxide nanocomposites combine the features of metal oxide with the flexibility of a polymer matrix, leading to the development of multifunctional materials, which can broaden their utilization range in different technical applications, including optoelectronic devices, solar cells, energy storage devices, nanodielectrics and photocatalytic materials [3,6,7,8,9,10]. The uniform dispersion of metal oxide nanoparticles within the polymer matrix leads to high-performance materials with improved properties, such as conductivity, low band gap energy and thermal stability [11]. In addition to the improvement in the above-mentioned characteristics of metal oxide/polymer nanocomposites, a significant enhancement has also been observed in terms of dielectric parameters (dielectric constant and dielectric loss) after the incorporation of metal oxide nanoparticles into a polymer matrix [12,13]. Generally, the characteristics of metal oxide/polymer nanocomposites depend on the size, shape and distribution of inorganic nanoparticles in the polymer matrix, their crystallinity and their compatibility with the polymer matrix [14]. It is possible to control the features of these materials by choosing the nanoparticle type, their content or the manner of how nanoparticles are dispersed and interact with the polymer matrix in order to develop advanced technological applications of metal oxide/polymer nanocomposites.

Poly(vinyl pyrrolidone) (PVP) is widely utilized as a suitable host material for different nanoparticles due to its film-forming ability, good environmental stability, simple operability and reasonable electrical conductivity [15,16]. Also, PVP possesses a high amount of amorphous phase, is soluble in water and polar solvents, and has great thermal stability, high dielectric constant, good storage capacity, low toxicity and easy processability [16,17,18]. The presence of carbonyl and CH-N groups on its side chains assures the complex formation between PVP and different inorganic ions, thereby ensuring surface passivation and good dispersion in composites [15,19].

Transition metal oxide nanoparticles have gained intense interest as potential multifunctional materials owing to their special physicochemical properties in many fields, including catalysis, optoelectronics, energy storage, environmental protection and pharmaceutics [1,2,20,21]. Among these metal oxides, copper oxide (CuO) is one of the important p-type semiconductors with a relatively small band gap in the range of 1.2–2.4 eV, having a monoclinic structure [22,23,24,25]. CuO nanoparticles have found applications in gas sensors, photocatalysis, solar cells, capacity media, electronic devices and bacterial inactivation materials. Moreover, CuO has attracted attention as a smart material due to its excellent chemical stability, low production cost, non-toxic nature and ability to diffuse easily in polymers [16,22,26,27]. Many studies have been devoted to metal oxide-based polymer nanocomposites, but only a few reports deal with poly(vinyl pyrrolidone)–copper oxide nanocomposites [28,29,30,31].

The present work aims to obtain nanocomposites based on PVP embedded with different amounts of copper oxide nanoparticles. The impact of CuO nanoparticles on the structural, optical and dielectric characteristics of the obtained nanocomposites was investigated by X-ray diffraction, scanning electron microscopy, electronic absorption spectra and dielectric measurements.

## 2. Experimental Section

### 2.1. Materials

The PVP used in this study was supplied by Fluka (Steinheim, Germany) (M_w_ = 360,000 g/mol). Copper sulfate, dimethylformamide (DMF) (99%) and sodium hydroxide were acquired from Sigma-Aldrich (St. Louis, MO, USA). Deionized water was employed during the preparation of the solutions.

### 2.2. Preparation of CuO Nanoparticles

Copper oxide nanoparticles were obtained using the chemical precipitation procedure as presented in ref. [24]. Briefly, 1 g of CuSO_4_ was dissolved in 50 mL of deionized water and stirred for 80 min at room temperature. Then, 1 M NaOH solution was added dropwise to the solution, with adjustment of pH to 14, and the mixture was stirred for 180 min at room temperature. The precipitate was washed several times with deionized water, dried in a vacuum oven at 80 °C for 5 h, and then calcined at 500 °C for 4 h.

### 2.3. Preparation of Film Samples

PVP and the obtained nanosized copper oxide were utilized as raw materials to make polymer films. PVP/CuO nanocomposite films were fabricated by the solution casting method. For this, 8 g of PVP was dissolved in 100 mL of DMF under magnetic stirring at 65 °C for 4 h until a homogeneous solution was obtained after cooling to ambient temperature. The resulting solution was separated into five equal parts. Copper oxide nanoparticles in different amounts (0.016, 0.033, 0.5, 1.0 and 2.0 wt%) were added to the PVP solutions and ultrasonicated at 65 °C for 45 min. The samples were labeled as PC1, PC2, PC3, PC4 and PC5. Also, a pure PVP film (P0) was obtained under the same conditions. Then, these solutions were poured onto glass Petri dishes for solvent evaporation at room temperature, and polymer films in the form of dry plane sheets were produced. The prepared films’ thickness was between 0.2 and 0.3 mm.

### 2.4. Characterization

Structural studies were performed using XRD measurements with a Bruker 18 Avance X-ray diffractometer (Bruker AXS, Karlsruhe, Germany) using CuK_α_ radiation source in the Bragg range of 10° ≤ 2θ ≤ 90°, with an accelerating voltage of 40 kV and a current of 40 mA. Particle morphology and elemental composition of the samples were analyzed with a Verios G4 UC scanning electron microscope (Thermo Scientific, Brno, Czech Republic) equipped with an energy-dispersive X-ray spectroscopy analyzer (Octane Elect Super SDD detector, Mahwah, NJ, USA). For SEM investigation, the samples were fixed on aluminum stubs with double-adhesive carbon tape and coated with a 6 nm platinum layer using a Leica EM ACE200 Sputter coater (Vienna, Austria) to provide electrical conductivity and to prevent charge buildup during exposure to the electron beam. The morphological study was carried out using a secondary electron detector ETD (Everhart–Thornley detector). The SEM micrographs were produced using an acceleration voltage of 5 kV and a spot size of 0.4 nA. For EDX analysis, the samples were analyzed using an acceleration voltage of 20 kV and a spot size of 6.4 nA. The ultraviolet–visible absorption spectra were obtained using a SPECORD210 Plus spectrometer (Analytik, Jena, Germany) in isopropanol solutions. A Shimadzu UV-3600 spectrometer equipped with an integrating sphere was employed to assess the band gap energy data. Dielectric spectroscopy measurements were performed using a Novocontrol Concept 40 broadband dielectric spectrometer (Novocontrol Technologies GmbH, Montabaur, Germany) featuring an Alpha-A frequency analyzer. Dielectric spectra were captured under isothermal conditions across a frequency range from 1 Hz to 1 MHz. The temperature ramp ranging from −150 °C to 150 °C was managed with the Quatro Cryosystem device. The samples, initially prepared as free-standing films, were positioned between two plated electrodes, and the measurements were conducted in a dry nitrogen atmosphere.

## 3. Results and Discussion

**Figure 1:**
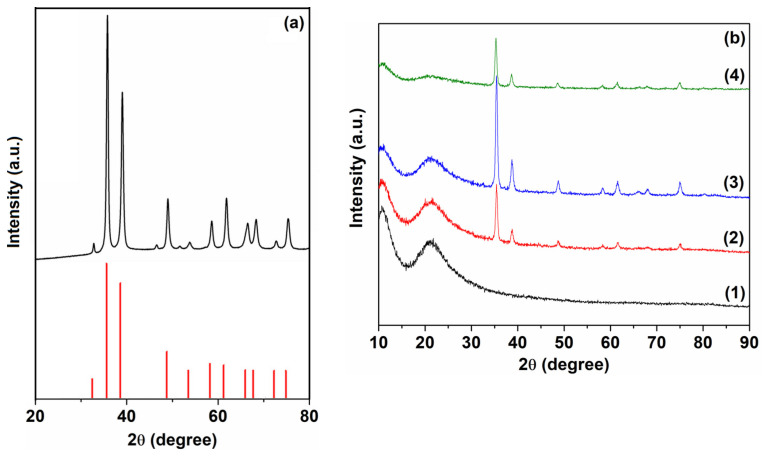
XRD patterns of (**a**) CuO and (**b**) (1) P0, (2) PC3, (3) PC4 and (4) PC5.

The XRD patterns of CuO, PVP and PVP/CuO nanocomposites at different loading levels are illustrated in Figure 1. As shown in Figure 1b, the PVP presents two broad diffraction peaks centered at Bragg angles of 22° and 11°, which reveal the predominantly amorphous nature of the polymer [32,33]. No other diffraction peaks were observed, confirming the amorphous feature of the PVP polymer matrix. The diffraction data of CuO nanoparticles (Figure 1a) indicate that these diffraction peaks can be assigned to the monoclinic structure of cupric oxide nanoparticles, which is in good agreement with previous results (JCPDS card No. 96-901-5569) [24,34]. The average crystallite size (D) of CuO nanoparticles and the PVP/CuO nanocomposites were estimated by the Scherrer equation, $D=0.9\lambda /\left(\beta cos\theta \right)$ [35], where λ denotes the X-ray wavelength, β is the full width at half maximum (FWHM) and θ is the diffraction angle for maximum intensity. The values of the crystallite sizes are presented in Table 1, and they fall in a range close to the values corresponding to CuO nanoparticles. Also, the microstructural strain, ε, was determined according to the relation $\varepsilon =\beta /\left(4tg\theta \right)$ [36]. Upon the incorporation of CuO nanoparticles, the intensity of the PVP amorphous peaks decreases significantly, whereas the intensity of the crystalline peaks increases, except for sample PC5 (Figure 1b). One can notice that CuO nanoparticles maintain their crystalline nature in the polymer matrix without any modifications in their positions. Also, the microstrain does not change very much as the nanoparticle level increases. The presence of X-ray signals due to the presence of CuO nanoparticles in the diffractograms of the PVP nanocomposites suggests that these crystalline nanoparticles are dispersed in the polymer matrix. Practically, the intensity of the main diffraction peaks in the PVP nanocomposites increases linearly with the CuO level, indicating a certain level of homogeneity in the dispersed nanoinclusions. The crystallinity percent, C, was determined according to the formula $C=A_{c}/\left(A_{c}+A_{a}\right)\times 100$, where A_c_ and A_a_ are the amorphous haloes and areas of crystalline diffraction peaks. The increase in the crystallinity percent can be due to the interfacial contacts between the polymer chain and CuO nanoparticles. The increased intensity of the diffraction peaks in the nanocomposites can be determined based on the fact that CuO nanoparticles act as a nucleating agent for the nanoparticle agglomeration, leading to an increase in the crystallinity percent [37].

**Figure 2:**
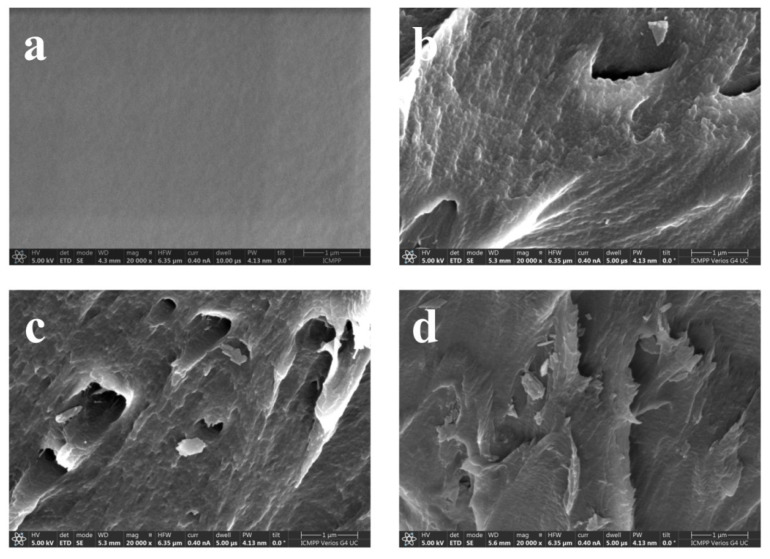
SEM micrographs of (**a**) P0, (**b**) PC3, (**c**) PC4 and (**d**) PC5.

The results of the SEM morphological analysis of the PVP samples and PVP/CuO nanocomposites are presented in Figure 2. The EM images were acquired from the fracture cross-section of the nanocomposite films. Figure 2a shows the micrograph of the PVP control sample. The micrograph of the PVP control sample exhibits a smooth, compact surface that is specific to polymers without a certain structure. As can be seen from the SEM micrographs (Figure 2b–d), CuO nanoparticles are rather homogeneously dispersed in the PVP matrix, and the increase in the percentage from 0.5 to 2 wt% does not indicate the presence of nanoparticle aggregations. In the case of the PVP/CuO composite materials, a certain texturing of the materials due to the presence of nanoparticles was observed. The micrographs of all PVP/CuO samples show that CuO nanoparticles are dispersed homogeneously in the polymer matrix. Regarding composite density, as the CuO nanoparticle loading increased from 1 wt% to 2 wt%, an increase in the density of the PVP/CuO composites was noticed. Thus, the sample with the highest percentage of CuO nanoparticles has the most homogeneous, compact appearance. The SEM image of the PVP shows the amorphous structure of the polymer, which is in agreement with the XRD data.

**Figure 3:**
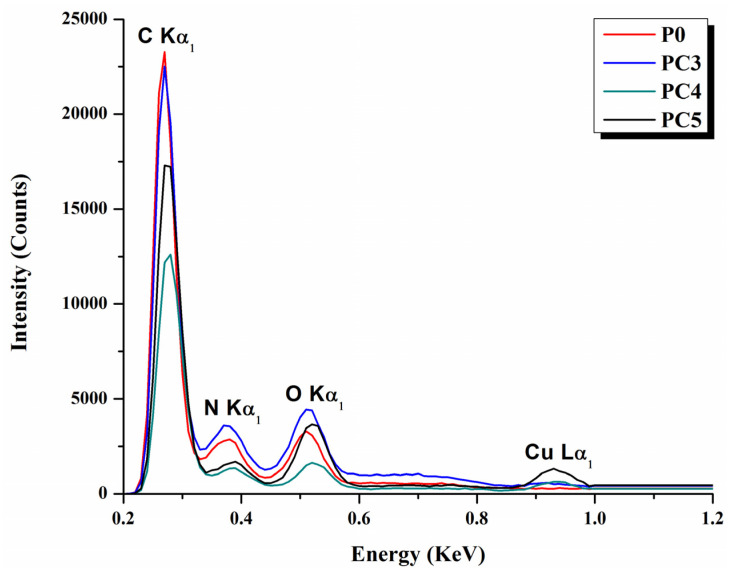
EDX spectra of PVP and PVP/CuO samples.

To highlight the elemental composition, EDX spectra of the composite materials were recorded. As can be seen in Figure 3, the spectrum shows the characteristic peaks of copper (K-shell transition energies with CuK peak at Lα 0.776 keV), carbon (K-shell transition energies with CK peak at Kα 0.277 keV), oxygen (K-shell transition energies with OK peak at 0.525 keV), and nitrogen (K-shell transition energies with NK peak at Kα 0.392 keV). In addition to these, we could observe the presence of platinum (M-shell transition energies with PtM peak at 2.048 keV), which comes from the coverage required for the examination. The PVP sample, as can be seen from Table 2, contains only C, N and O. The PVP/CuO samples present Cu, with the mass percentage varying from 1.3% in PC3 to 11% in PC5. These results confirm the incorporation of CuO nanoparticles inside poly(vinyl pyrrolidone) chains.

**Figure 4:**
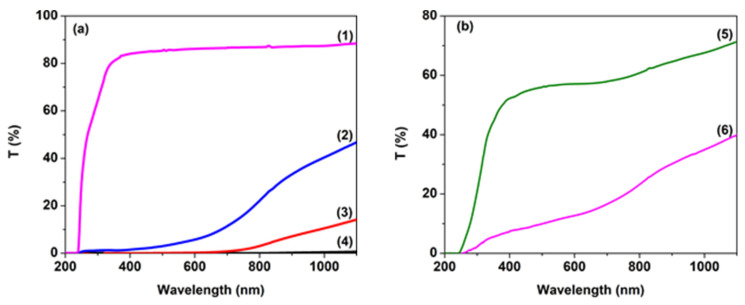
UV–Vis transmission spectra of PVP nanocomposites embedded with CuO nanoparticles: (**a**) (1) P0, (2) PC3, (3) PC4 and (4) PC5; (**b**) (5) PC1 and (6) PC2.

**Figure 5:**
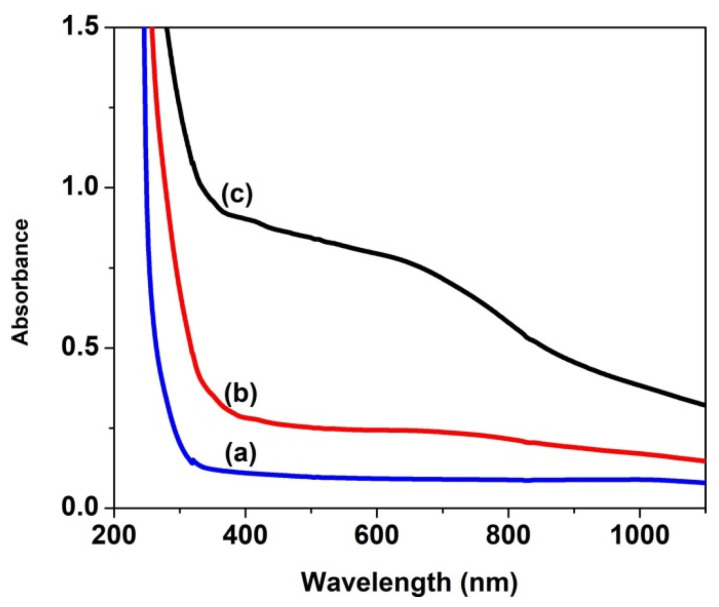
UV-Vis absorption spectra of pure PVP and PVP/CuO nanocomposites: (**a**) P0, (**b**) PC1 and (**c**) PC2.

The ultraviolet–visible absorption spectra provide important information for understanding the band structure, optical behavior, band gap energy and optical parameters in the crystalline and non-crystalline materials. Figure 4 and Figure 5 display the electronic absorption spectra of the PVP nanocomposite films at several loading levels. It is observed that all of these films present strong absorption in the UV range (below 350 nm), and their transmittance decreases significantly in the longer wavelength range as the copper oxide content increases in the polymer film. After adding 2% of CuO nanoparticles to the polymer matrix, the film transmittance reaches 1% (Figure 4a). It is evident that the transmission characteristics of the PVP/CuO films differ significantly as a function of the CuO level in the nanocomposites. With a low CuO content (0.016%) the transmittance reaches around 60% at 700 nm, while the transmittance of the PVP film containing 0.033% CuO decreases sharply to 20%, and the impact of incorporation of CuO nanoparticles on changes in the film transmission behavior is clearly observed (Figure 4b). These results suggest that due to the incorporation of CuO in the composites and the high decrease in film transmission, these materials can be used as protective layers against sunlight. Also, as the CuO content in the PVP matrix increases, the transmittance edge shifts to longer wavelengths (Figure 4) due to the increased charge transfer between the metal oxide nanoparticles and PVP matrix, leading to important changes in the band structure of the material [38]. Moreover, an increase in the absorption of the PVP/CuO nanocomposite films can be determined based on modifications in the packing density of polymer chains containing different amounts of CuO nanoparticles.

**Figure 6:**
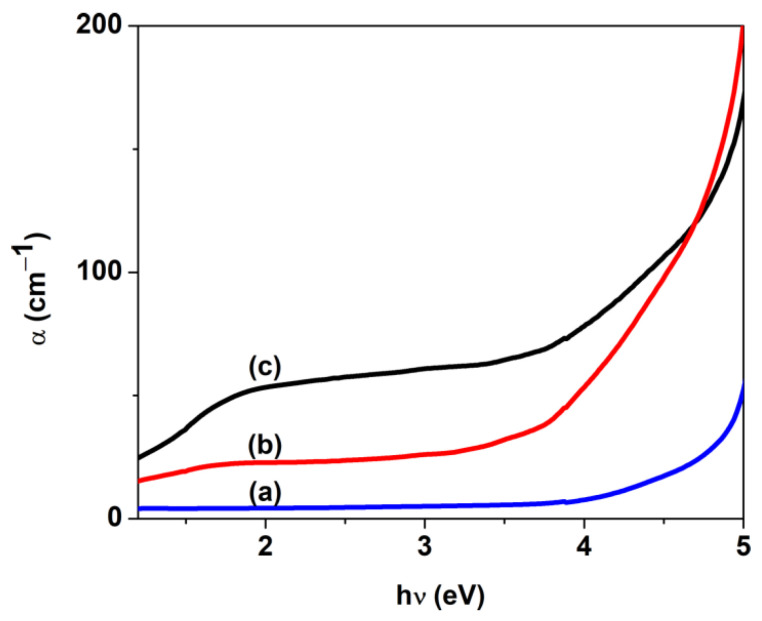
Absorption coefficient of PVP/CuO nanocomposites versus hν: (**a**) P0, (**b**) PC3 and (**c**) PC4.

Taking into account the relation between the intensity of incident light (I_0_) and the intensity of transmitted light (I), the absorption coefficient, α, can be determined using the following formula:

(1) α=2.303A/d

where A denotes the absorbance ($A=lg\left(I_{0}/I\right)$), and d is the film thickness. The incorporation of CuO nanoparticles in the PVP matrix leads to an increase in the absorption coefficient values, which can be 10 times higher compared to a pure PVP film (Figure 6). This increase of the absorption coefficient can be due to a higher light energy loss in the films via scattering and absorption.

**Figure 7:**
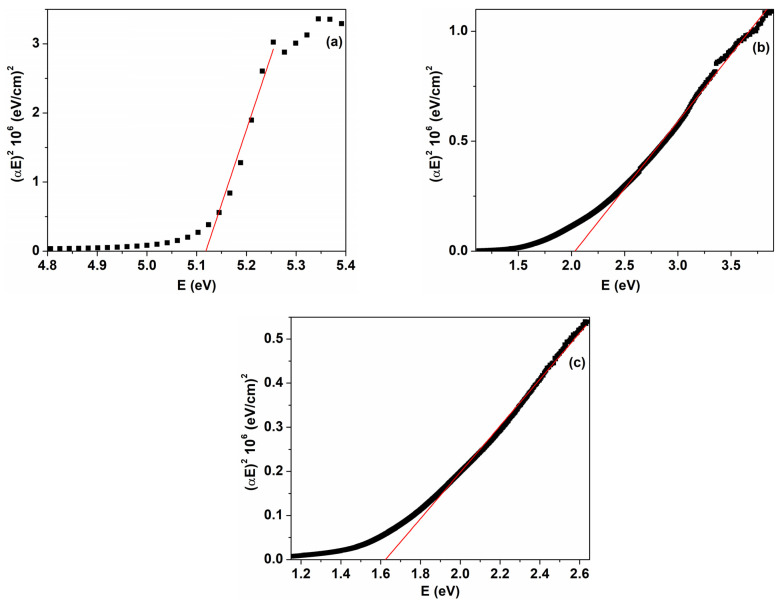
(αhν)^2^ as a function of photon energy (hν) for PVP/CuO films: (**a**) P0, (**b**) PC3 and (**c**) PC4. The black squares are experimental data and red line corresponds to the linear part of the plot used to calculate optical parameters.

**Figure 8:**
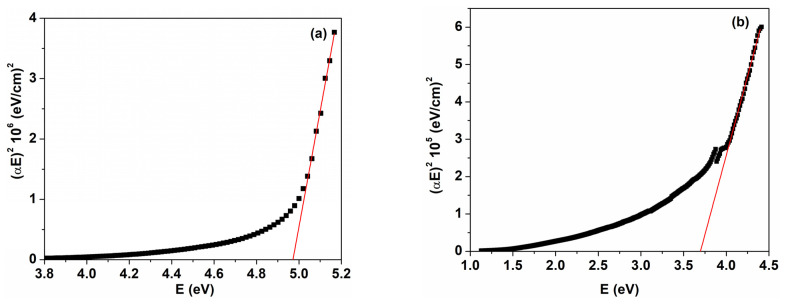
Variation in (αhν)^2^ with photon energy for two PVP/CuO nanocomposites: (**a**) PC1 and (**b**) PC2. The black squares are experimental data and red line corresponds to the linear part of the plot used to calculate optical parameters.

The optical band gap energy (E_g_) can be estimated from the UV-Vis absorption data using the Tauc relation [39,40]:

(2) $${\left(\alpha h\nu \right)}^{n}=A\left(h\nu -E_{g}\right)$$

where α represents the absorption coefficient; hν is the incident photon energy; A is a constant; and n is a parameter that identifies the type of optical transition, with the value n = 1/2 for direct allowed transition and n = 2 for indirect allowed transition. The optical band gap values were obtained from the intercept of the linear part of the plot (αhν)^1/n^ as a function of photon energy with the hν axis. Figure 7 reveals the variation in ${\left(\alpha h\nu \right)}^{2}$ as a function of hν for the PVP/CuO nanocomposites. The value corresponding to the direct energy gap for the pristine PVP film is around 5.12 eV, which is close to most reported data [15,19]. The calculated E_g_ values for the CuO nanoparticle-loaded PVP films were estimated to be 2.03 eV (PC3) and 1.62 eV (PC4) (Figure 7). As shown in Figure 7 and Figure 8, the E_g_ values decrease as the content of CuO nanoparticles increases in the polymer matrix. In consequence, the E_g_ values diminish from 4.97 eV for PC1 to 1.62 eV for PC4. A value of 3.70 eV was obtained for the PVP/CuO nanocomposite containing 0.033% CuO (PC2) (Figure 8, Table 1). This response can be explained by the interaction between the CuO nanoparticles and PVP host chains, leading to the generation of localized defect states in the optical band, and their density increases with higher metal oxide levels. Consequently, the energy band gap is reduced due to the fact that electrons can overpass from the maximum of the valence band to the minimum of the conduction band [19,33,41]. In this way, band gap engineering can be appropriately performed by adding specific loadings of CuO nanoparticles into polymer films. A similar behavior of E_g_ versus metal oxide loading was reported previously [19,22,33,42,43].

The Urbach tail energy, E_U_, is related to the defect levels or localized energy states within the band gap. The relation between the absorption coefficient, α, and the Urbach energy is illustrated by the equation: $\alpha =\alpha_{0}exp\left(h\nu /E_{U}\right)$, where α is the absorption coefficient calculated according to Equation (1) and $\alpha_{0}$ is a constant [44,45]. After linearization of the above-mentioned equation, the following relation is obtained:

(3) $$ln\alpha =ln\alpha_{0}+h\nu /E_{U}$$

**Figure 9:**
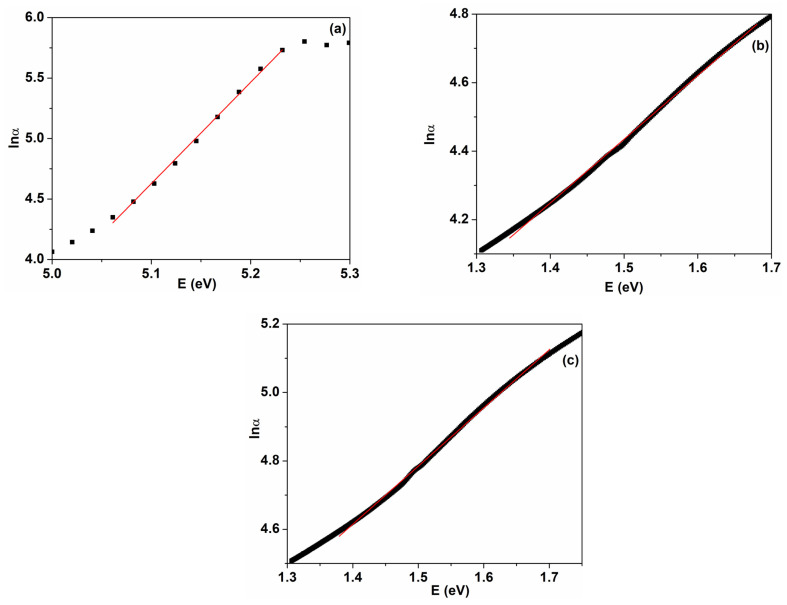
Urbach plots for PVP/CuO nanocomposites: (**a**) P0, (**b**) PC3 and (**c**) PC4.

By plotting lnα as a function of the photon energy, hν=E, the E_U_ for the pure and PVP/CuO nanocomposite films can be determined from the reciprocal of the slopes of the linear part, as shown in Figure 9. The values of the Urbach energy are listed in Table 1. It is evident that E_U_ increases from 119.4 meV, the value estimated for the pristine PVP, to 537.7 meV and 588.2 meV, respectively, for the nanocomposite samples with a CuO content of 0.5% and 1.0%. The variation in the Urbach energy as a function of the CuO content indicates the introduction of defects in the band structure of the materials. The introduction of metal oxide nanoparticles in the polymer matrix results in additional defect states in the polymer host, leading to irregularities in the polymer chain packing due to the PVP–nanoparticle interactions [46,47]. Also, it is evident that the values of the Urbach energy increase while the energy gap decreases, indicating a higher content of defects, which is in agreement with previous results [48,49].

The determination of the refractive index of materials is very important for optoelectronic and electronic applications or optical device fabrication. The refractive index can be estimated from the values calculated for the optical band gap using the following relation [50]:

(4) n2−1/n2+2=1−Eg/2012

Based on the estimation of the refractive index values shown in Table 1, it is noticed that this optical parameter increases nonlinearly as the CuO loading increases in the nanocomposites. This increase in the refractive index can be due to the changes in the packing density of the material, leading to a decrease in the interatomic spacing [46].

**Figure 10:**
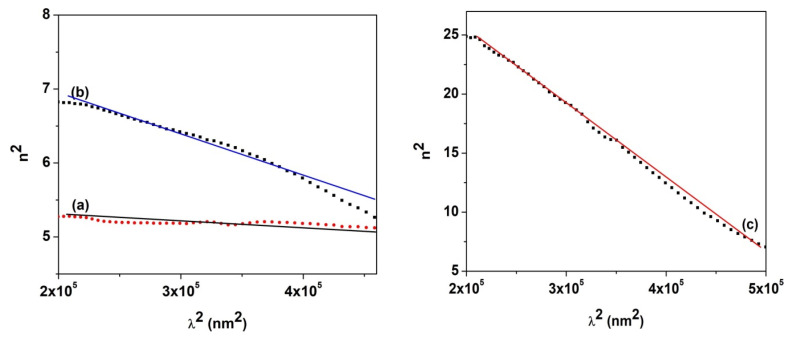
Variation in n^2^ with the square of the photon wavelength (λ^2^) for PVP/CuO nanocomposites: (**a**) P0, (**b**) PC3 and (**c**) PC4.

Also, two essential optical parameters, the ratio of carrier concentration to electron effective mass, (N/m^•^), and the high-frequency dielectric constant (ε_∞_), can be evaluated using the dispersion relation [51,52]:

(5) $$n^{2}=\varepsilon_{\infty }-\left(\frac{e^{2}}{\pi c^{2}}\right)\left(\frac{N}{m}\right)\lambda^{2}$$

where e represents the electron charge, c is the light velocity, and m^•^ is the effective mass of the carrier. The parameters N/m^•^ and ε_∞_ can be determined from plotting n^2^ as a function of λ^2^ (Figure 10), where the ratio (N/m^•^) is estimated based on the slope of the straight line and ε_∞_ is calculated from the extrapolation of the linear part to λ^2^ = 0. The value of the ratio (N/m^•^) increases as the CuO content increases in the nanocomposites, suggesting the growth of free charge carriers, while the values of the gap energy decrease. The values of ε_∞_ are greater than the refraction index, confirming that the presence of free charges carriers in the nanocomposites contributes to the polarization process [41].

**Figure 11:**
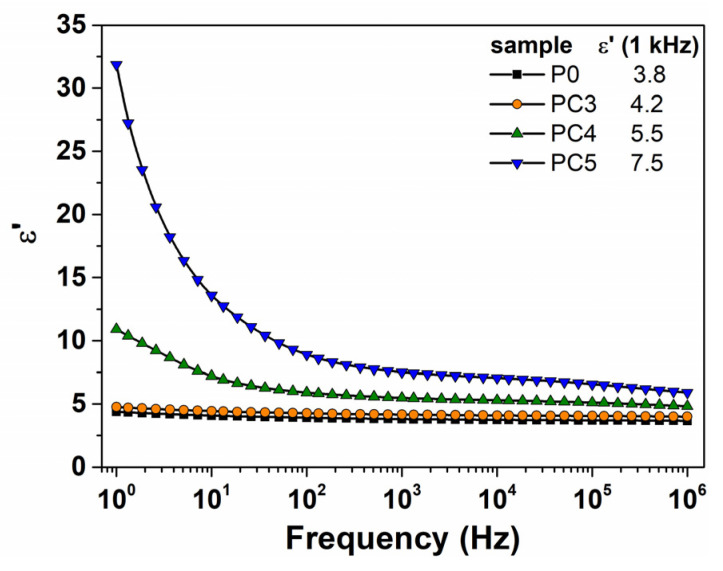
Evolution of dielectric constant with alternating electrical field frequency for all the investigated samples. The ε′(f) spectra were obtained at room temperature. Numerical values of ε′ derived at 1 kHz are presented in the figure.

Metal oxide/polymer nanocomposites can be regarded as good media for applications in a wide range of capacitive devices. The dielectric response of the PVP/CuO nanocomposites was analyzed by dielectric spectroscopy. Figure 11 provides a comparative illustration of the evolution of dielectric constant (ε′) with changes in frequency for the pristine PVP and CuO-based nanocomposites. It is evident that the PVP matrix exhibits the lowest value of ε′ with a slight tendency to decrease further as frequency increases. The dielectric polarization magnitude is attributed to the contribution of the permanent dipole moments from the repeating units of the host polymer, specifically the carbonyl C=O groups. Upon the introduction of CuO nanoparticles into the PVP matrix, a gradual enhancement in the dielectric polarization occurs in the nanocomposites. For the 0.5% CuO nanocomposite, there is a slight increase in the ε′ magnitude, and the behavior of the dielectric constant with changes in the frequency is similar to that of the host matrix (Figure 11). In contrast, higher CuO levels lead to remarkable polarization, which is particularly noticeable at low frequencies. Further, the ε′ values present a significant decline as the frequency increases till low constant values are found in the high frequency range. This enhancement in the dielectric constant with an increase in the metal oxide level in the PVP films can be attributed to the electrostatic interactions between the CuO nanoparticles and dipolar groups of the PVP chain, leading to additional polarization under an electric field [53,54]. For example, at 1 kHz, the ε′ for the PVP is 3.8, whereas for the CuO/PVP nanocomposites, the following values were found: 4.2 (PC3), 5.5 (PC4) and 7.5 (PC5). Also, this behavior was only observed at low frequencies, which can be indicative of an electrode polarization-type effect, probably arising from the agglomeration of charge carriers at the interface between the sample under investigation and the electrodes used for the dielectric measurements [55]. Moreover, the presence of interfacial polarization suggests a rather good homogenization in the PVP matrix, which is in agreement with the SEM data. The agglomeration of CuO nanoparticles can result in hindrance in the polarization response to the applied field, leading to lower values of the dielectric constant [56]. We observed that at a low frequency (1 Hz), the magnitude of the dielectric constant exhibits a high value, namely 11 for sample PC4 and 32 for sample PC5. The values reported in the literature for the dielectric constant of PVP or PVP-based blends that incorporate metal oxide nanoparticles are lower [15,27,53,57]. At higher frequencies, the values of the dielectric constant remain practically independent of the frequency due to the effect of charge accumulation in the nanocomposite films [58].

**Figure 12:**
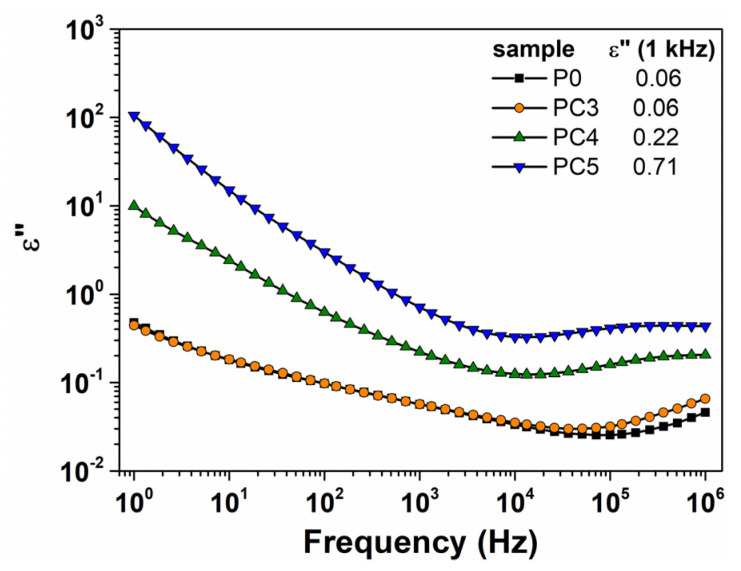
Variation in dielectric loss with frequency at room temperature for pristine and CuO-based PVP nanocomposites.

The variation in the dielectric loss (ε″) versus frequency for the PVP/CuO nanocomposites with different contents of CuO nanoparticles is depicted in Figure 12, which shows that the dielectric losses are very high at low frequencies. Both the PVP matrix and the nanocomposite containing 0.5% CuO demonstrate minimal losses across the entire frequency range, indicative of the dipolar relaxation processes inherent to these materials. It was noticed that the evolution of dielectric loss of the as-prepared nanocomposites presents the same trend observed in the dielectric constant graphs up to 10^4^ Hz. As the CuO level in the nanocomposites increases, a noticeable increase in ε″ was observed at low frequencies. This process can be attributed to the influence of CuO nanoparticles, which act as generators of charge carriers and obscure the dipolar relaxation signal of the polymer backbone, with the increased accumulated charges leading to the polarization of the polymer/metal oxide interfaces. Furthermore, the dielectric loss shows a wide minimum in the frequency range of 10^4^–10^5^ Hz, and then a slight increase in ε″ values were found for the samples (Figure 12).

**Figure 13:**
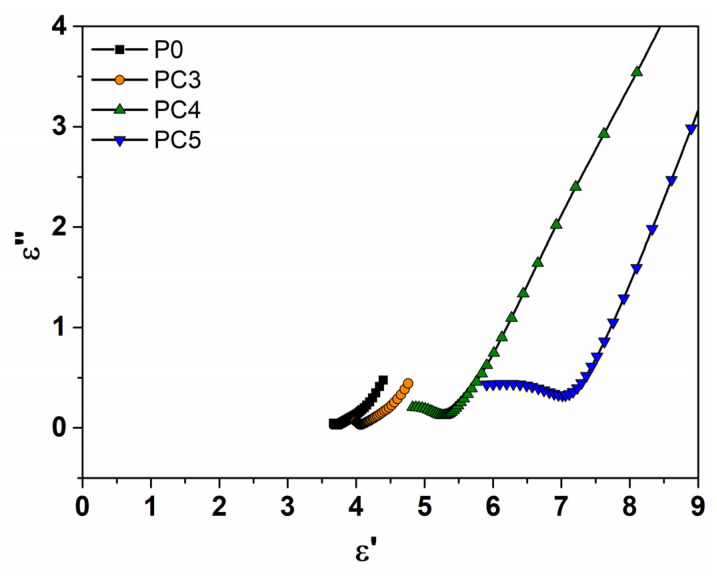
Cole–Cole plots of ε″ vs. ε′ at room temperature for nanocomposite films with different CuO dosages.

The Cole–Cole plots of ε″ in relation to ε′ are presented in Figure 13. The plots show incomplete semicircular arcs centered below the ε′ axis for the nanocomposites with the higher CuO loadings. These incomplete arcs are shifted to higher frequencies with an increase in the CuO level. The deviation from the semicircular arcs can be related to the increased ac conductivity, and the right parts of the Cole–Cole graphs suggest the presence of dc conductivity and interfacial polarization [26,59], as confirmed by the conductivity data. It was revealed that the direct optical energy gap decreases, whereas conductivity increases, as the metal oxide content increases in the nanocomposites, which is in agreement with previous results [13].

**Figure 14:**
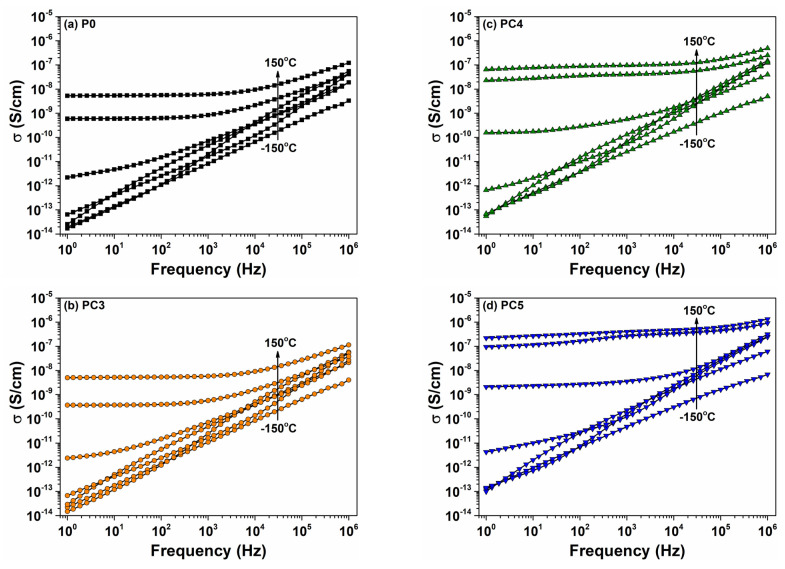
Evolution of conductivity with frequency at various temperatures for (**a**) P0, (**b**) PC3, (**c**) PC4 and (**d**) PC5.

Figure 14 displays the isothermal graphs depicting conductivity (σ) as a function of frequency at specific temperature values for all the as-prepared films. At low temperatures, a linear increase in conductivity with frequency is evidenced, indicative of the ac conductivity effect associated with bonded charge carriers. At higher temperatures, a conductivity plateau emerges due to the dc conductivity component, which is attributed to free charge carriers within the nanocomposites. As temperature increases, the flat region rises with low frequencies and is enlarged with higher frequencies. It is worth noticing that the signal of dc conductivity is dominant in the σ(f) dependencies for nanocomposites PC4 and PC5 (Figure 14c,d), aligning closely with the isothermal plots of the ε′ and ε″ dielectric parameters. In the obtained nanocomposites, the highest values of electrical conductivity at 100 °C were observed for the nanocomposites with higher CuO loadings, which were found to be 10^−8^ S/cm for PC4 and 10^−7^ S/cm for PC5. The incorporation of CuO nanoparticles increases the samples’ crystallinity, assuring facile routes for charge carrier movements in this way.

**Figure 15:**
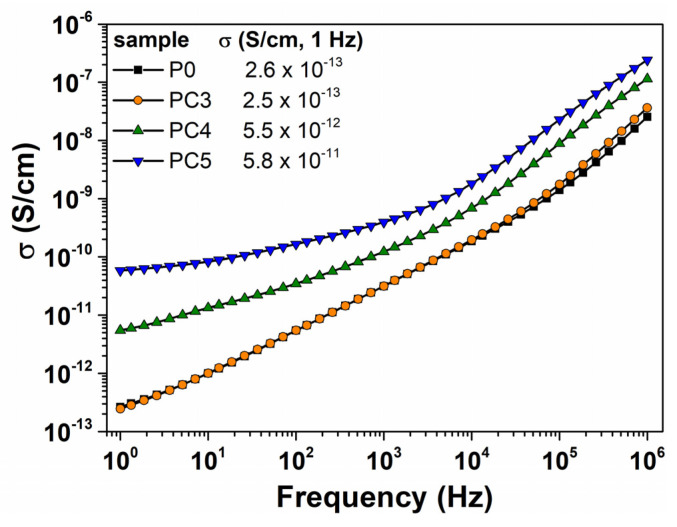
Variation in electrical conductivity with frequency at room temperature for all the investigated samples.

The plots of electrical conductivity versus frequency for the PVP matrix and PVP/CuO materials at room temperature are presented in Figure 15. The dielectric signal of the PVP matrix is managed by a linear increase in σ as frequency increases, a common behavior that is specific for dielectric materials lacking free charge carriers. The PC3 nanocomposite displays a similar pattern to the pure PVP matrix, suggesting that the low content of CuO nanoparticles is insufficient to facilitate charge carrier transport through the polymer host. In the case of the nanocomposites containing 1 wt% and 2 wt% CuO, the dielectric signal, a characteristic of the dc conductivity component, exhibits an increase at low frequencies, indicating the presence of free charge carriers in the nanocomposites. Based on the numerical values of conductivity shown in Figure 15, conductivity is significantly enhanced by two orders of magnitude when 2 wt% of CuO is incorporated into the PVP host.

**Figure 16:**
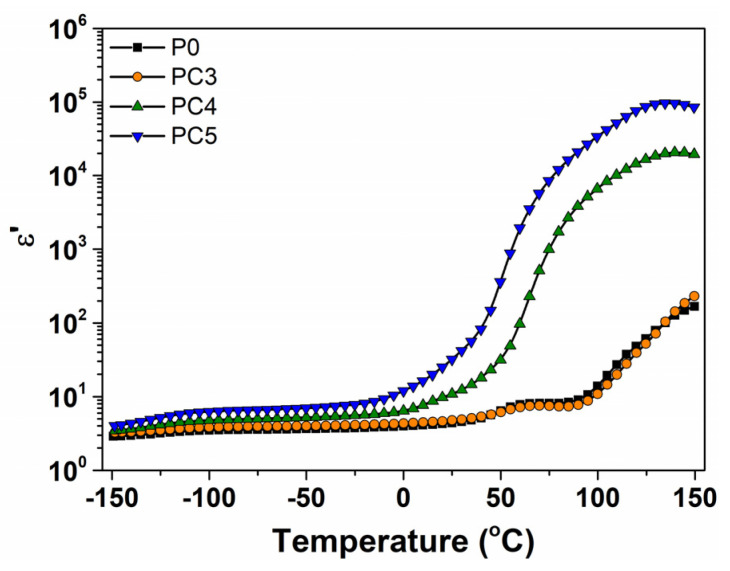
Temperature-dependent dielectric constant values at 1 Hz for PVP/CuO nanocomposites.

The temperature dependencies of the dielectric constant for the CuO/PVP nanocomposites at 1 kHz are illustrated in Figure 16. It is noted that ε′ exhibits a gradual increase with rising temperatures, a common trend observed for polymer materials. It is evident that the ε′(T) dependencies of the PVP matrix and the nanocomposite PC3 are practically similar. At low temperatures, ε′ presents a temperature-independent behavior. A step increase in ε′ is detected around 50 °C and can be associated with a dipolar relaxation process related to the corresponding peak, probably due to an α-relaxation process [60]. By increasing the temperature, the polymer viscosity decreases and the dipoles acquire sufficient energy to follow the applied electric field, confirming the thermally activated dielectric polarization of the polymer film. As temperature increases, a substantial enhancement in ε′ was observed as a function of CuO loading. The significant increase in the dielectric constant at higher temperatures (>50 °C) suggests that charge carriers can move freely in the polymer sample due to the segmental mobility of the polymer chains enhancing the dipolar orientation, and hence, the dielectric constant increases [54,60]. Also, the density of charge carriers is higher when the CuO nanoparticle level in the host matrix increases, leading to their accumulation at the interface between the polymer and CuO nanoparticles; thus, higher values of ε′ were obtained.

**Figure 17:**
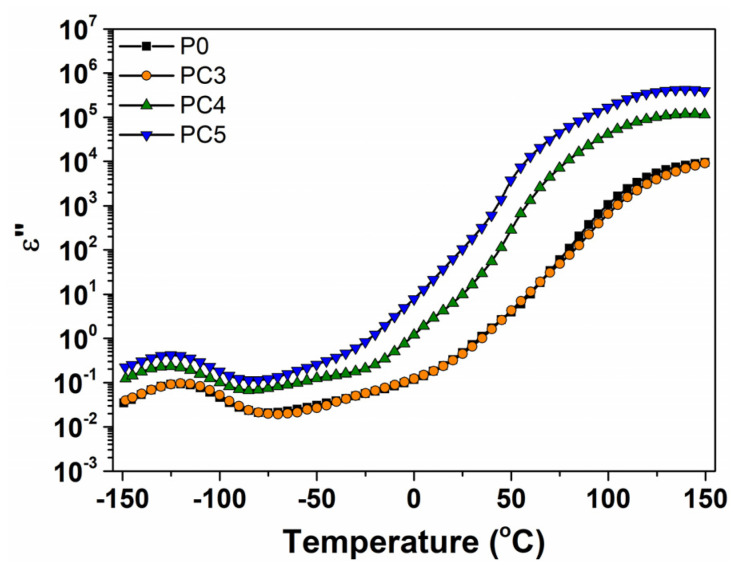
Dielectric loss as a function of temperature at 1 Hz for PVP loaded with different amounts of CuO nanoparticles.

Figure 17 demonstrates the temperature dependence of dielectric loss for the pristine PVP and nanocomposites at 1 kHz. It can be noted that two distinct signals were found for these samples. Thus, ε″(T) exhibits a dipolar relaxation peak arising from the molecular fluctuations of dipoles (discernible in the low temperature range between −150 °C and −75 °C) and a second peak in the high temperature range due to ionic relaxation associated with charge conduction (at temperatures higher than 0 °C). The incorporation of CuO nanoparticles in the host matrix also improves the values of dielectric loss.

**Figure 18:**
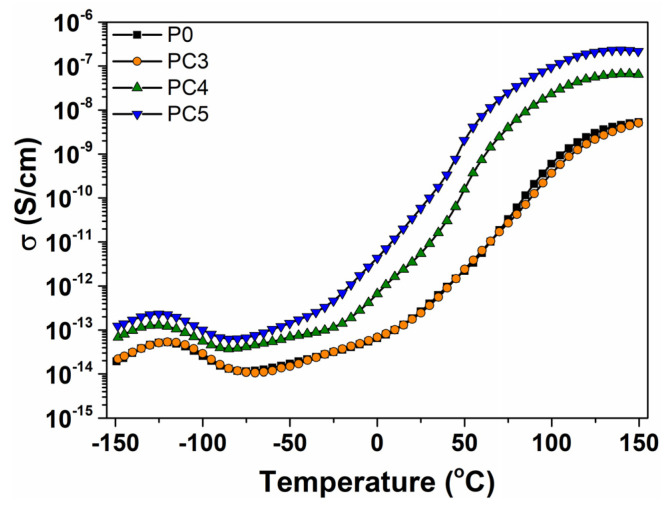
Evolution of conductivity with temperature at 1 Hz for PVP/CuO nanocomposites.

The isochronal plots of conductivity (Figure 18) depict processes analogous to those observed in ε″(T). Furthermore, it is noteworthy that the nanocomposites with 1 wt% and 2 wt% CuO exhibit a higher magnitude of conductivity across the entire temperature range.

## 4. Conclusions

This paper reports the structural, optical and dielectric characteristics of polymer nanocomposites derived from PVP and CuO nanoparticles. PVP/CuO nanocomposite films were obtained by the solution casting procedure, which is eco-friendly, cost-effective and simple, using different weight percentages of CuO nanoparticles in the polymer matrix. The appearance of the diffraction peaks of CuO nanoparticles in XRD traces of the samples, and their increase in intensity as the CuO level increases confirms the formation of the PVP/CuO nanocomposites. The UV-Vis analysis revealed a concentration-dependent decrease in optical band gap, with values dropping from 5.12 eV for the PVP host matrix to 2.03 and 1.62 eV, respectively, for the nanocomposites containing 0.5% and 1.0% CuO. In addition, the Urbach energy of the PVP film was evaluated to be 119.4 meV, and this value increases upon the incorporation of CuO nanoparticles into the polymer matrix. The dielectric constant of the nanocomposite films decreases with increasing frequency, whereas the CuO loading leads to an increase in the dielectric constant at room temperature. The frequency-dependent conductivity of the PVP/CuO nanocomposites shows a linear increase at low temperatures due to the ac conductivity associated with bonded charge carriers. In summary, the properties of these metal oxide nanocomposites can be controlled by CuO nanoparticle loading.

## Figures and Tables

**Table 1:** Structural parameters of PVP/CuO nanocomposites.

Sample	D (nm)	ε × 10^3^	d (Å)	E_g_ (eV)	E_U_ (meV)	n	N/m
CuO	17.24	1.69	2.525				
PC3	17.87	6.38	2.533	2.03	537.7	2.0045	0.52 × 10^−6^
PC4	18.55	6.15	2.531	1.62	588.2	2.9223	1.39 × 10^−6^
PC5	17.82	6.42	2.542	1.08		3.3030	3.79 × 10^−6^
P0				5.12	119.4		

**Table 2:** Elemental composition for the obtained PVP/CuO nanocomposites based on the EDX spectra.

Sample	P0		PC3		PC4		PC5	
	At%	Wt%	At%	Wt%	At%	Wt%	At%	Wt%
C K	57.9	50.3	49.0	40.6	59.1	47.6	54.9	44.5
O K	25.8	26.2	32.4	31.4	26.1	24.5	20.0	18.9
N K	16	18.5	17.7	19.6	12.8	13.7	22.4	24.2
Cu K	–	–	0.3	1.3	1.3	5.7	2.6	11.0
PtM	0.4	5.0	0.5	7.1	0.7	8.6	0.1	1.4

## Data Availability

Data present in this study are available from the corresponding author upon request.
